# Plastic water bottle detection model using computer vision in aquatic environments

**DOI:** 10.1038/s41598-025-09300-8

**Published:** 2025-07-10

**Authors:** Andrew Heller, Matthew Jacobs, Gilberto Acosta-González, Anna Basola, Jessica Beck, Wesley Garnes, Jarelys A. Hernández Molina, Alanso Johnson, Rebecca Kiriazes, Melissa Lenczewski, Ellen O’Brien, Grace Pooley Deans, Rhea Roxy, Blaise Trapani, Jason H. Davison

**Affiliations:** 1 Catholic University of America, Department of Electrical Engineering and Computer Science, Washington D.C., 20064 United States; 2 SECIHTI – Unidad de Ciencias del Agua, Centro de Investigación Científica de Yucatán A.C. (CICY), Cancún, 77500 Mexico; 3 Catholic University of America, Department of Civil and Environmental Engineering, Washington D.C., 20064 United States; 4 University of Puerto Rico Mayagüez Campus, Department of Geology, Mayagüez, 00681 United States; 5 University of Maryland, Department of Aerospace Engineering, College Park, 20740 United States; 6 Northern Illinois University, Institute for the Study of the Environment, Sustainability and Energy, DeKalb, 60115 United States

**Keywords:** Computer vision, Macroplastics, Water bottle, River, Environmental sciences, Environmental impact, Hydrology

## Abstract

Watershed macrotrash contamination is difficult to measure and requires tedious and labor-intensive processes. This work proposes an automated approach to waste counting, focusing on using computer vision, deep learning, and object tracking algorithms to acquire accurate counts of plastic bottles as they advect down rivers and streams. By using a combination of several publicly available labeled trash and plastic bottle image datasets, the model was trained to achieve high performance with the YOLOv8 object detection model. This was paired with the Norfair object tracking library and a novel post-processing algorithm to filter out false positives. The model performed extremely accurately over the test scenarios with just one false positive and recalls in excess of 0.947.

## Introduction

Geoscience researchers are leveraging machine learning (ML) and artificial intelligence (AI) algorithms to address a wide range of challenges^1–6^. In the field of water resources, AI and ML techniques have opened new research pathways including water management models based on Markov Decision Process, water depth analysis with Convolutional Neural Networks, and modeling infiltration rates with the Firefly Algorithm^7–9^. One popular approach, known as computer vision, extracts information from images or video and has widespread application in medical sciences, autonomous vehicles, and manufacturing. Computer vision offers advantages like low-cost video recording sensors, real-time analysis, and a data-rich input.

Computer vision has also found application in trash detection and classification. For example, a deep convolutional neural network was used to detect and classify roadway waste images recorded by a vehicle-mounted camera. As one of the first trash studies, the researchers collected and annotated nearly 20,000 images for their application^10^. To enhance the limited availability of training data, a trash debris dataset was developed with over 48,000 annotations^11^. Other trash datasets include the Trash Annotations in Context (1,500 images), The Plastic Bottles in the Wild (8,000 images), The Unmanned Aerial Vehicle - Bottle Dataset (25,000 images), and The Images of Waste (9,000 images)^12–15^.

Previous iterations of trash-detecting software have focused on general solutions capable of detecting multiple trash types^16^. Some of these tools have been applied in waste sorting systems including recycling stations^17–19^. However, these tools are not easily adaptable to the outdoor environment because they rely on cameras close to the objects, have controlled environmental conditions (e.g., lighting and background) and use a limited set of test items. Several general-purpose tools have been developed for watershed trash detection^20–27^. Yet, these tools encounter key challenges like the absence of counting algorithms, lower accuracy for detecting distant objects, and high false positives rates.

This project aims to address these limitations by developing a specialized tool that only counts plastic bottles in aquatic ecosystems as they move across the video’s frame. The program, referred to as **botell.ai**, implements an object detection model that finds the presence and location of a bottle within a frame^28^. **botell.ai** then matches each detected item and tracks their movement. In the final stage of post-processing, the tracked objects are analyzed for motion, and stationary items are filtered out from the model. Additionally, this project follows Integrated, Coordinated, Open, Networked (ICON) best practices with a publicly available source code, input video files, and output model result videos^29^.

Quantifying macroplastic debris using software like **botell.ai** is essential for addressing plastic pollution in aquatic ecosystems. Water bottles are a common source of plastic debris, frequently found as pollutants worldwide^30,31^. Over time, these bottles degrade, contributing to the growing presence of microplastics and nanoplastics in both freshwater and marine environments^32–35^.

## Methods

**Fig. 1:**
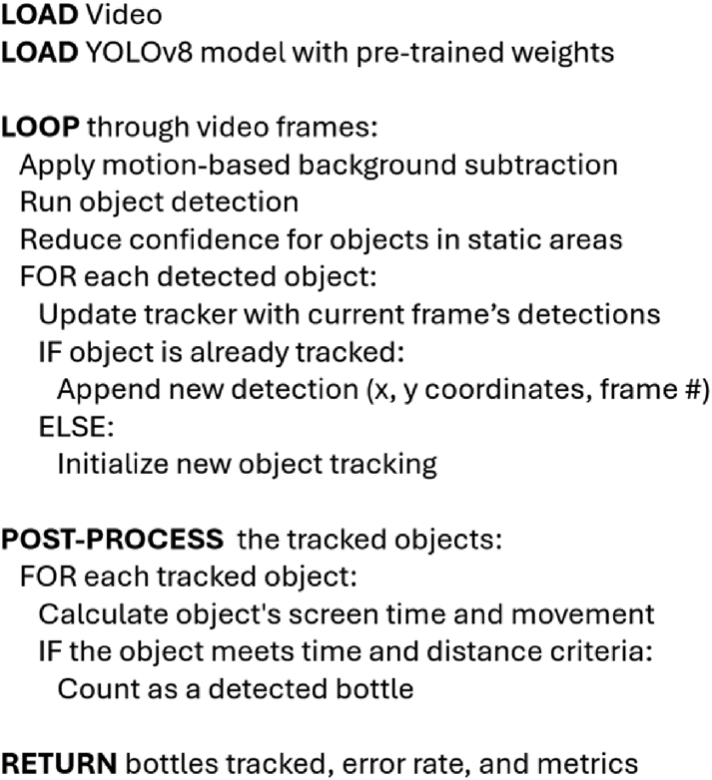
Flow chart of the detection and tracking software process.

The bottle detecting application combines computer vision with object tracking to count plastic bottles floating downstream. The process begins with training of YOLOv8, an object detection model. Field videos are then processed by the trained model, which identifies and logs each bottle location and unique ID. Next, Norfair, a tracking algorithm, links the detections across frames and calculates velocity and overall movement. Finally, a post-processing script refines the output. The pseudo-code for this model is shown in Fig. 1.

### Object Detection

YOLO (You Only Look Once) is a popular and effective real-time object detection and classification model^36^. The algorithm combines object localization and class prediction steps into a single network, and predicts both simultaneously. The model simultaneously implements a global class probability map, for predefined cell sizes as a grid over the image, and creates bounding boxes with probabilities. The object detection is simplified by framing as a regression analysis rather than a multi-step classification. The YOLO framework is a fast-performing model, which is accessible and executable on diverse hardware platforms. Processing times with YOLO may exceed real-time video playback, especially with modern graphics processing units (GPU).

Since its inception, YOLO has undergone many iterations both as a research tool^37^ and in commercial applications^38^. This project uses YOLOv8 a state-of-the-art model developed by the Ultralytics team^39^. While leveraging the speed and simplicity of the YOLO architecture, YOLOv8 is a collection of models of increasing complexity (from simplest to most complex nano, small, medium, large, and extra large), as well as a host of built-in preprocessing and configuration options. The included image augmentation library optimizes the input dataset by rotating, manipulating colors, and transforming images to create a more effective and larger training dataset.

YOLOv8 is used in the **botell.ai** program to first identify the plastic bottles floating downstream and assigns a label ID. It then calculates a confidence score for each item ranging from 0 (lowest probability) to 1 (highest likelihood), and creates a bounding box with pixel location. This information is then passed on to the next step, Norfair, for object tracking and counting.

### Object Tracking

Norfair^40^ is a customizable lightweight Python library for real-time multi-object tracking. Norfair is used in conjunction with compatible detection software, like YOLOv8 to produce tracking metrics based on the given object detection. Norfair uses a Kalman filter for motion estimation, similar to DeepSORT, and includes extensive customization and visualization toolbox^41^.

The bottle detecting tracking algorithm was designed for a top-down view, as found when mounting a camera on a bridge or walkway over the waterway. This increases the likelihood that the bottles are continuously tracked and avoids potential occlusions from waves, floating debris, or other obstructions. The code is developed as a flexible tool and can also be used with cameras mounted along a stream bed or on a drone, with reduced robustness.

### Detection Filtering

**Fig. 2:**
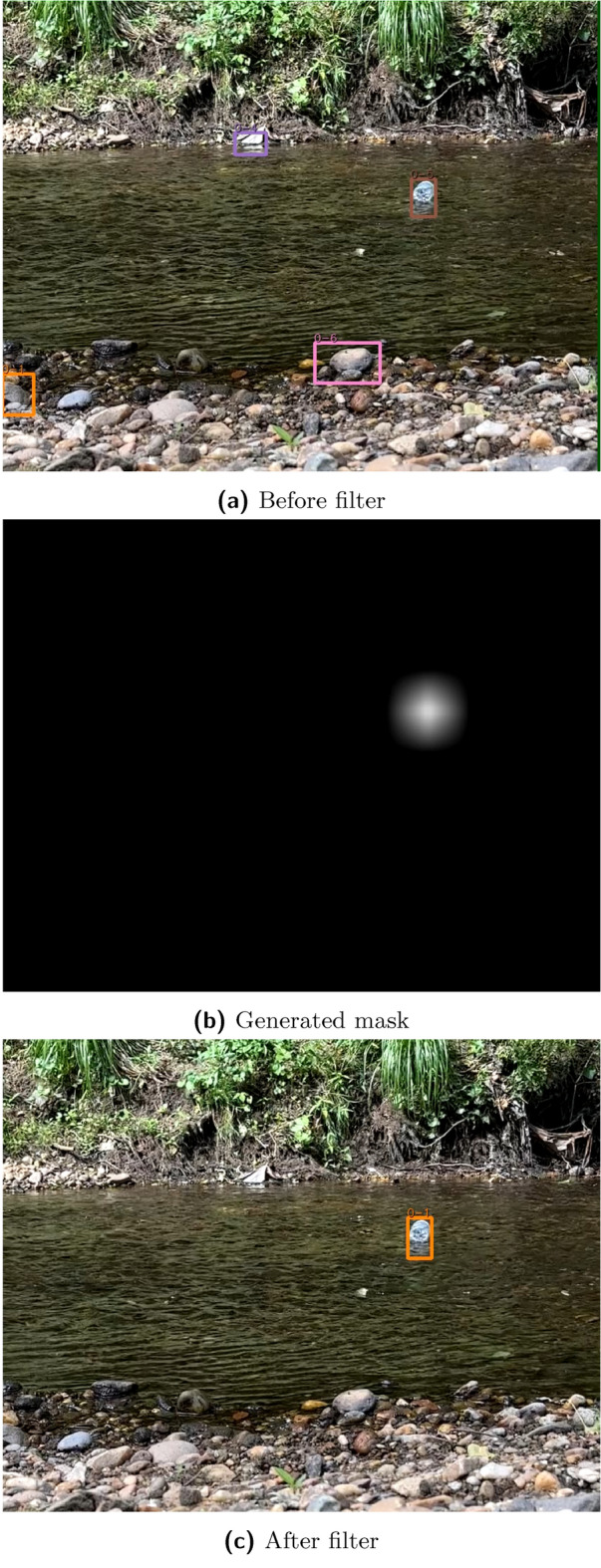
Filtering process which includes the (**a**) before filter detections, (**b**) the generated mask, and (**c**) the post-filtering detections. The filter decreases the number of detections from four (1 true positive and 3 false positives) to one (1 true positive).

The detection filtering is set up into two separate stages. The first phase, shown in Fig. 2, implements OpenCV’s Gaussian mixture model for background removal^42^. The filter creates a binary mask of suspected moving objects and removes small noisy elements (leaves or small waves) with a morphological closing algorithm. A smoothing effect is then applied that turns the binary mask into a distribution of suspected movement. The mask is then normalized from -0.33 for no movement to 0 for expected motion. These values are finally added back to the detection’s confidence values.

The lower bound of -0.33 was selected based on testing to sufficiently penalize non-moving detections. Users can tune this value and the minimum confidence threshold for tracking based on their specific environment. In our testing, the background removal filter, combined with appropriate threshold tuning, consistently allowed for robust filtering of non-moving objects while preserving valid detections. Detection filtering is only applied when the camera remains stationary. For moving camera scenarios, such as drone applications, this technique cannot be used to blur the background.

The second, post-processing stage, analyzes the amount of time an object is tracked and the distance the object moves across the video frame. Typically, the majority of false positives have either an extremely short duration (i.e. one or two frames) or do not travel across the majority of the screen. These false positives are most likely noise (e.g. image disturbances from reflection or waves) rather than a bottle-like shape. They have little in common between frames, lack consistency in direction, and travel short distances. This additional filter increases the overall robustness of the model. The object time (number of frames) and distance traveled (across a frame) are both user controlled features.

## Training Data

The **botell.ai** model was trained, tested, and validated with 13,480 images from three publicly accessible datasets. The model was trained using 11,964 images (88.7%) and tested with 492 images (3.6%). It was then validated with 1,024 images (7.5%) to calculate overall accuracy and skill. The datasets were selected for their size and consistency in label quality and, in combination, include natural, staged, and synthetic data. This diversity enables the model to generalize across different camera types, environmental conditions, and visibility ranges. The three datasets include the following:

**racnhua**^43^ is a publicly available Roboflow-based dataset consisting of 9,889 images of plastic bottles. This dataset came pre-split into training, testing, and validation sets, containing 8415, 1011, and 505 bottle images respectively. Several images were converted from segmentation annotations to bounding boxes.

**The Unmanned Aerial Vehicle - Bottle Dataset**, or UAV-BD, is a large collection with more than 25 thousand images of bottles, and over 34 thousand instances of plastic bottles across the dataset^14^. Despite the high quality and quantity of the images in this data, the images have very low variability in background and lighting between each image. For this reason, the UAV-BD was reduced to approximately one third using a random selection process. This reduction prevents over fitting of the detection model with one type of image observation.

**Trash Annotations in Context**^12^, or TACO, is a set of 6,004 images of 18 different categories of trash and litter. Because many mislabels existed for the plastic bottles category, we filtered out any images containing plastic bottles, removed all annotations, and utilized this dataset solely to provide background images to reduce false positives.

## Training

The object detection model, YOLOv8, comes in a wide variety of configurations depending on the task and efficiency requirements of the application, ranging from YOLOv8n (nano) to YOLOv8xl (extra large). For initial testing and model development, this project used YOLOv8n (nano) and YOLOv8s (small) because they reduce training time during testing stages. The final project implemented the medium size, YOLOv8m, because of its balance between training time, inference speed, and model performance.

YOLOv8 defaults to an image input size of 640x640. This means that all images passed through the model are downsampled to this resolution before any processing takes place. Custom resolutions are possible, but were not needed due to the high quality precision and recall results. Model training was done on an NVIDIA RTX A4000, accompanied by an AMD Ryzen Threadripper PRO 5995WX (64-Cores) and 32GB of RAM.

The training model was set to single class mode and configured with a batch size of 32 and a maximum limit of 500 epochs. An epoch represents a complete pass through the entire training dataset, while a batch refers to the number of items processed by the model before updating gradients and parameters. All images were placed into a 2x2 mosaic in groups of 4. YOLOv8 applied four separate image augmentations to increase the variability of the training set including copy-paste (a random chance for an instance of an object to be pasted onto a different background), scaling (mosaic is scaled by a factor of a random number between 0.5 and 1.5), rotation (mosaic is rotated by a random number of degrees between 0 and 180), and flipping (mosaic has a random chance of being horizontally flipped).

## Validation

**Fig. 3:**
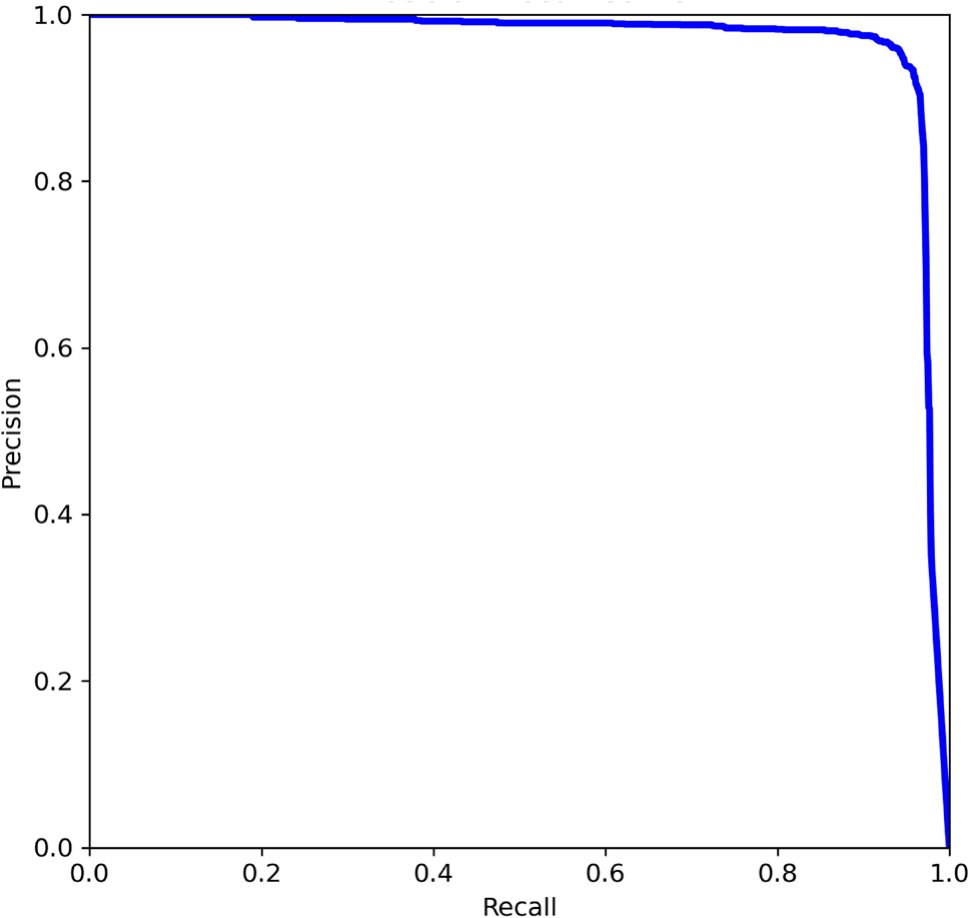
Final precision-recall curve of the model after training, where a larger area under the curve is better.

**Fig. 4:**
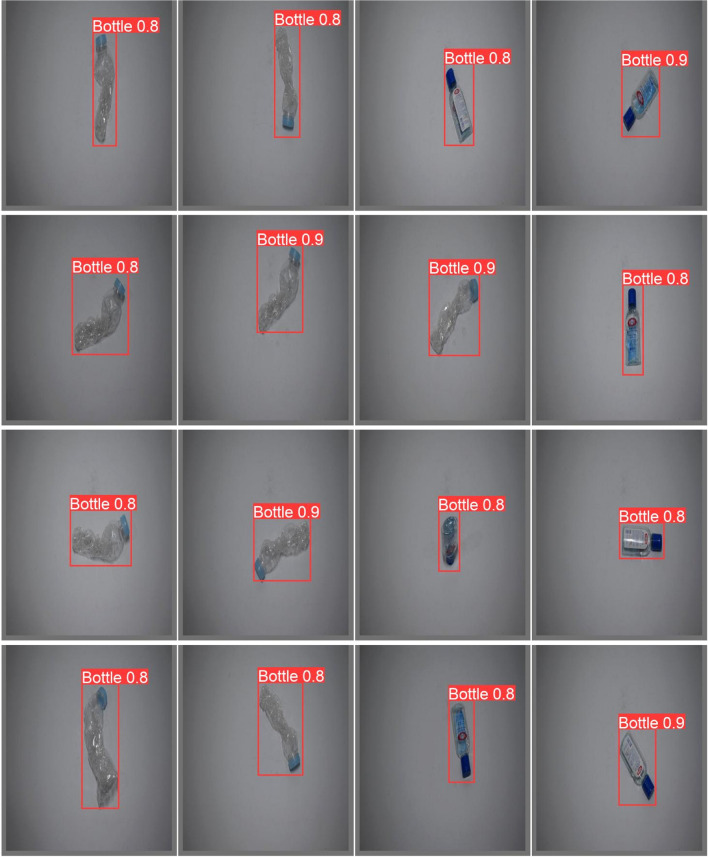
Detected bottles from the racnhua dataset indicating strong skill on incomplete or damaged bottles.

After each epoch, metrics are run, and are compared to the previous epochs. To prevent over-fitting, YOLOv8 implements patience, a parameter that stops training if the model’s performance on the validation set doesn’t improve after a set number of epochs, saving the best-performing weights. The **botell.ai** model was set to a patience value of 50. Training stopped at epoch 338, with the best performance on the validation set occurring at epoch 288. The final precision-recall curve (epoch 288) is shown in Fig. 3 and validation testing with confidence scores are shown in Fig. 4.

## Performance

The trained YOLOv8 model performs reliably across a wide range of conditions, provided that the bottle’s contour is visible. This holds true regardless of camera angle or water clarity, as long as there is a clear line of sight to the object’s edges. While lighting conditions do affect performance, the impact largely depends on the camera’s sensor quality. In low-light environments, limited light reaching the sensor can constrain detection accuracy. For optimal results, the model is best used in well-lit settings.

## Results and Discussion

**botell.ai** was tested under controlled conditions where researchers placed bottles in the environment. The first four trials were conducted in Sligo Creek, a small subwatershed in Maryland, using a GoPro HERO12 Black camera (1080p at 60 frames per second). Tests were carried out on a pedestrian bridge and atop a weir (a small dam), with each setup filmed from both optimal (top-view) and sub-optimal (30-degrees) angles. The dual-angle approach was used to assess the impact of camera positioning and evaluate the robustness of the algorithm.

The fifth test was also filmed in Sligo Creek, but used a cellphone camera attached to the side of the stream bed. The last test was done with a drone in Yucatán, Mexico. The results from the six tests are shown in Table 1. All bottles released into the environment were collected by researchers at the end of the experiment.

**Table 1:** Test results from the six test scenarios.

	Actual	True positives	False negatives	False positives	Recall
Bridge (optimal angle)	38	36	2	0	0.947
Bridge (sub-optimal angle)	38	12	26	0	0.316
Weir (optimal angle)	52	51	1	0	0.981
Weir (sub-optimal angle)	52	37	15	0	0.712
Sideview	7	7	0	0	1.0
Drone	6	6	0	1	1.0

### Pedestrian Bridge

The first location used two cameras placed on a pedestrian bridge. The cameras were placed about eight feet from the water surface, with the optimal direction pointing directly down at the water. The camera with the sub-optimal angle had a more shallow side view. After positioning the camera, the researchers released 38 bottles into the stream. The videos were then cropped and zoomed to an 1:1 aspect ratio.

**Fig. 5:**
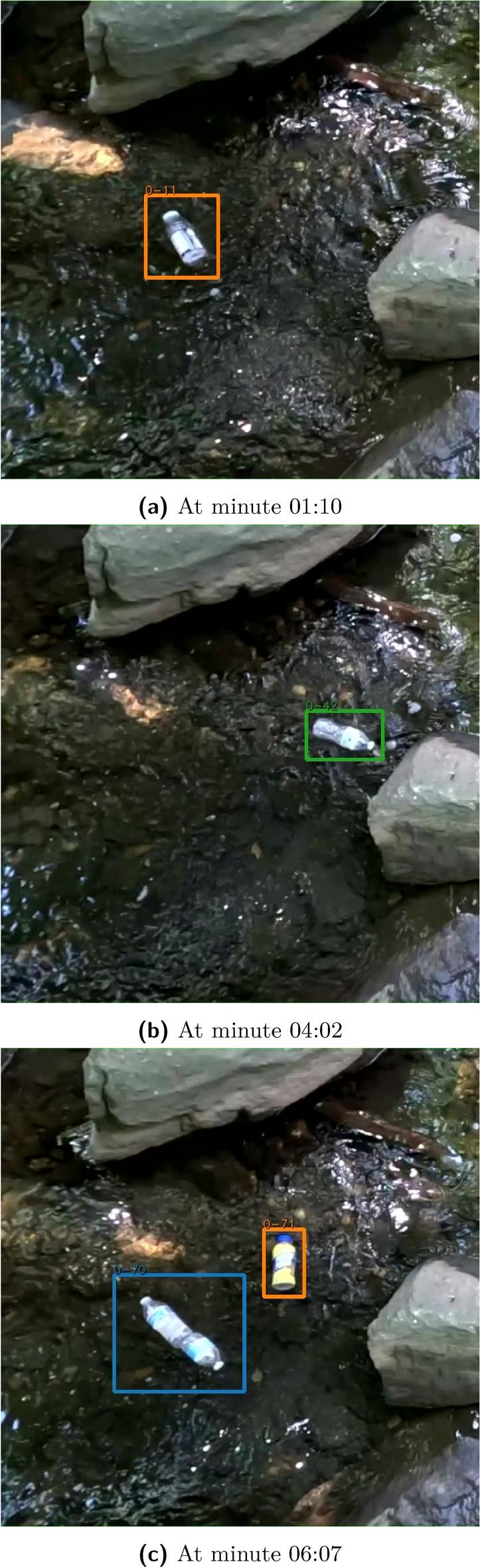
Pedestrian bridge filmed at the optimal angle. (**c**) contains an example of three bottles in the image. Only two bottles were detected, with one false negative resulting from two bottles touching and appearing as a single object.

**Fig. 6:**
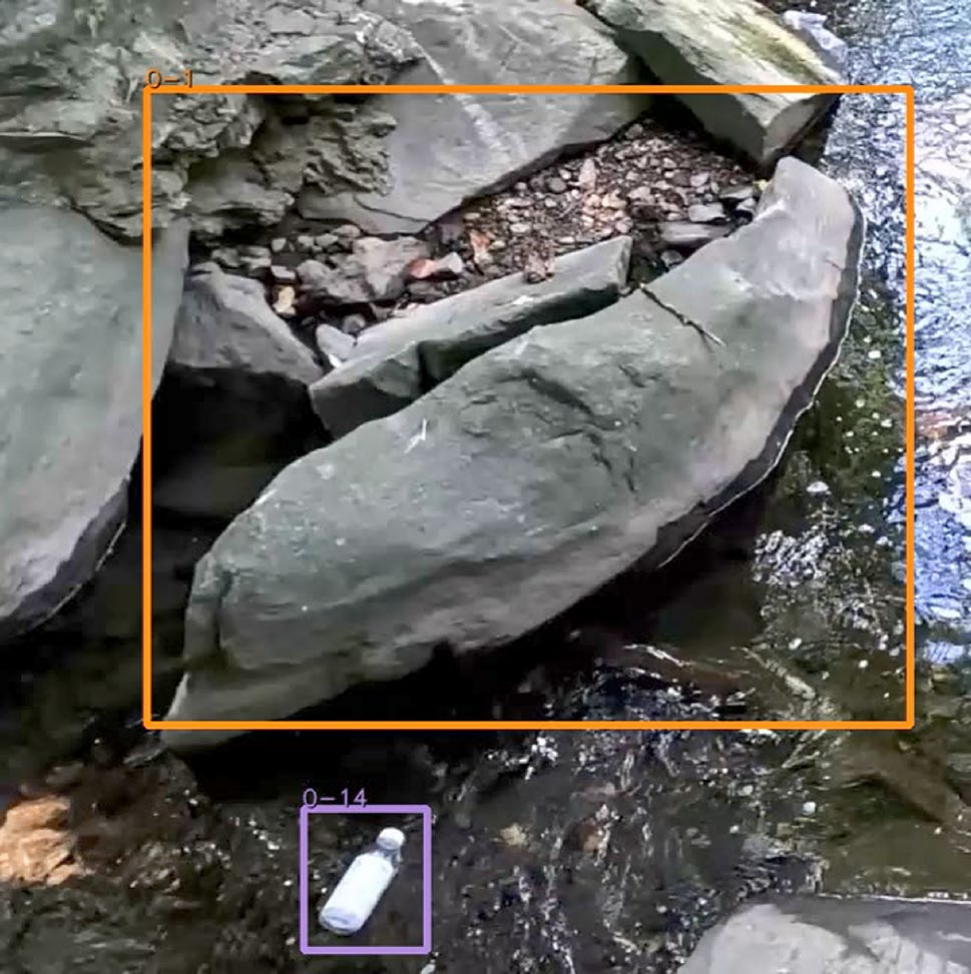
Bridge at the sub-optimal angle. The large boulder caused a tortuous path resulting in tracking issues. The boulder was detected with a low confidence, it was not counted by the algorithm because it does not move and it was only detected for few continuous frames.

The **botell.ai** algorithm counted 36 of the 38 bottles for the optimal angle test (recall of 0.95), with example frames shown in Fig. 5. One of the false negatives, bottom image of Fig. 5, was due to the two bottles connecting and floating downstream as a single item. The sub-optimal angle only counted 12 of the 38 bottles advecting downstream (recall of 0.32). The sub-optimal detection had many tracking issues due to the tortuous water path and drops in tracking, as seen in Fig. 6. The sub-optimal angle detected the large boulder as a potential bottle in this frame, but it was filtered out because it did not move and was not on screen past the threshold period.

### Weir

**Fig. 7:**
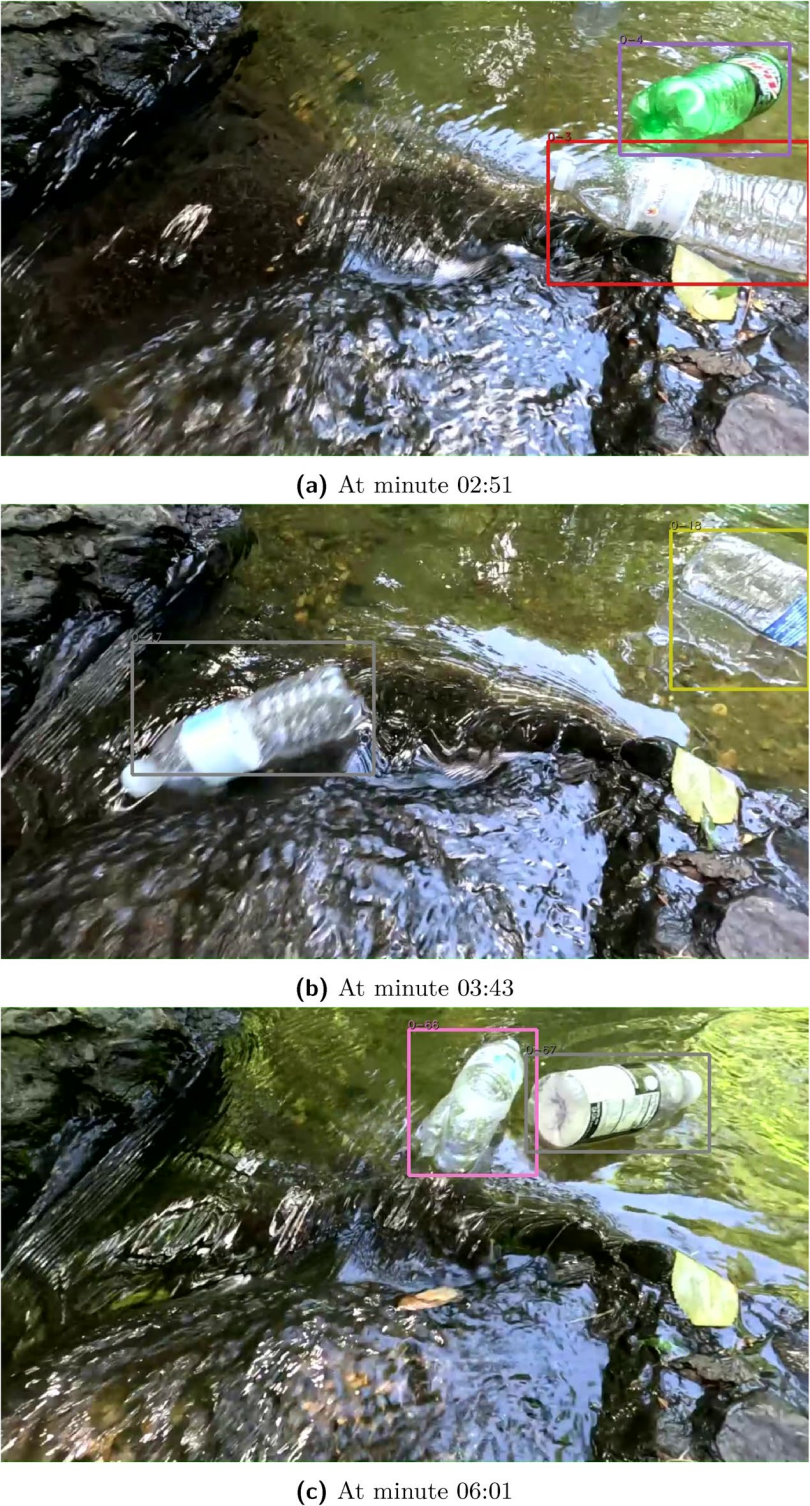
Weir test from the optimal angle. Bottles were accurately counted in this scenario because of the clear flow path, lighting, camera proximity.

**Fig. 8:**
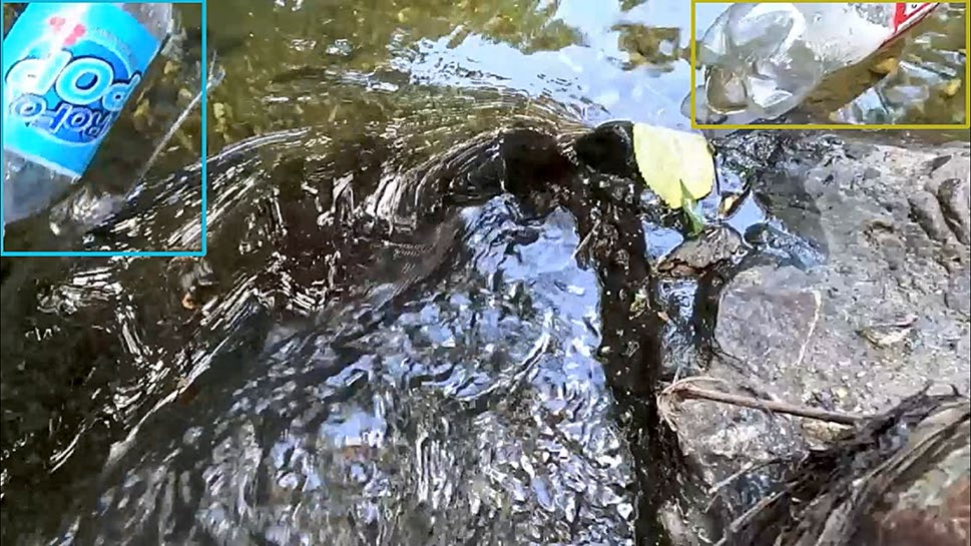
Weir at the sub-optimal angle. Placement was too close to the water surface for detection. The bottles did not stay in frame for an adequate period of time.

The second test location was filmed on top of a weir discharge, where water flowed over a narrow concrete opening. The cameras were stationed less than two feet away from the water’s surface. No video cropping or zooming was necessary in this test. The test counted 51 of the 52 bottles for the optimal angle (recall of 0.95), shown in Fig. 7. However, the sub-optimal only captured 37 of the 52 bottles (recall of 0.71), which was caused by the video being too close to the water surface, shown in Fig. 8. The weir test outperformed the pedestrian bridge video because the camera was closer to the water’s surface. Thus, a larger majority of the camera frame was comprised of bottles.

### Sideview

**Fig. 9:**
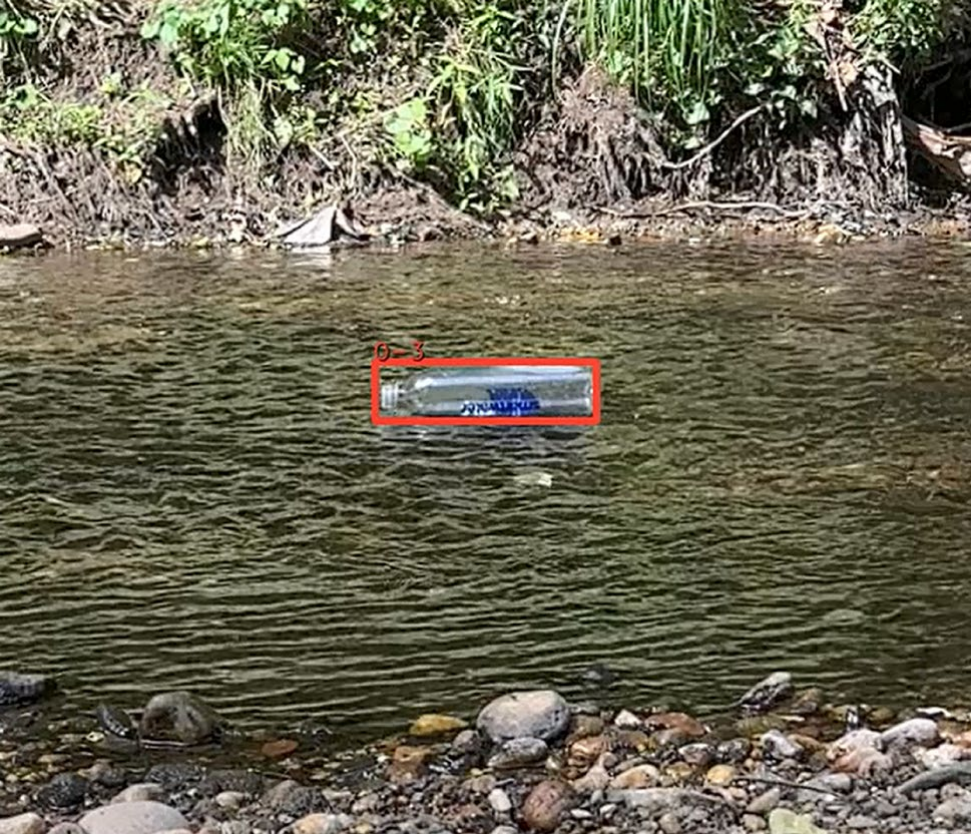
Stream sideview example with a single bottle detection.

The fifth test was completed on the side of the shore using a cellphone camera. The video included 7 bottles and the **botell.ai** program successfully counted all 7. There were no false positives or negatives and had a recall and precision of 1.0. An image from this test video is shown in Fig. 9. The bottles were relatively close to the camera and were easily detectable.

### Drone

**Fig. 10:**
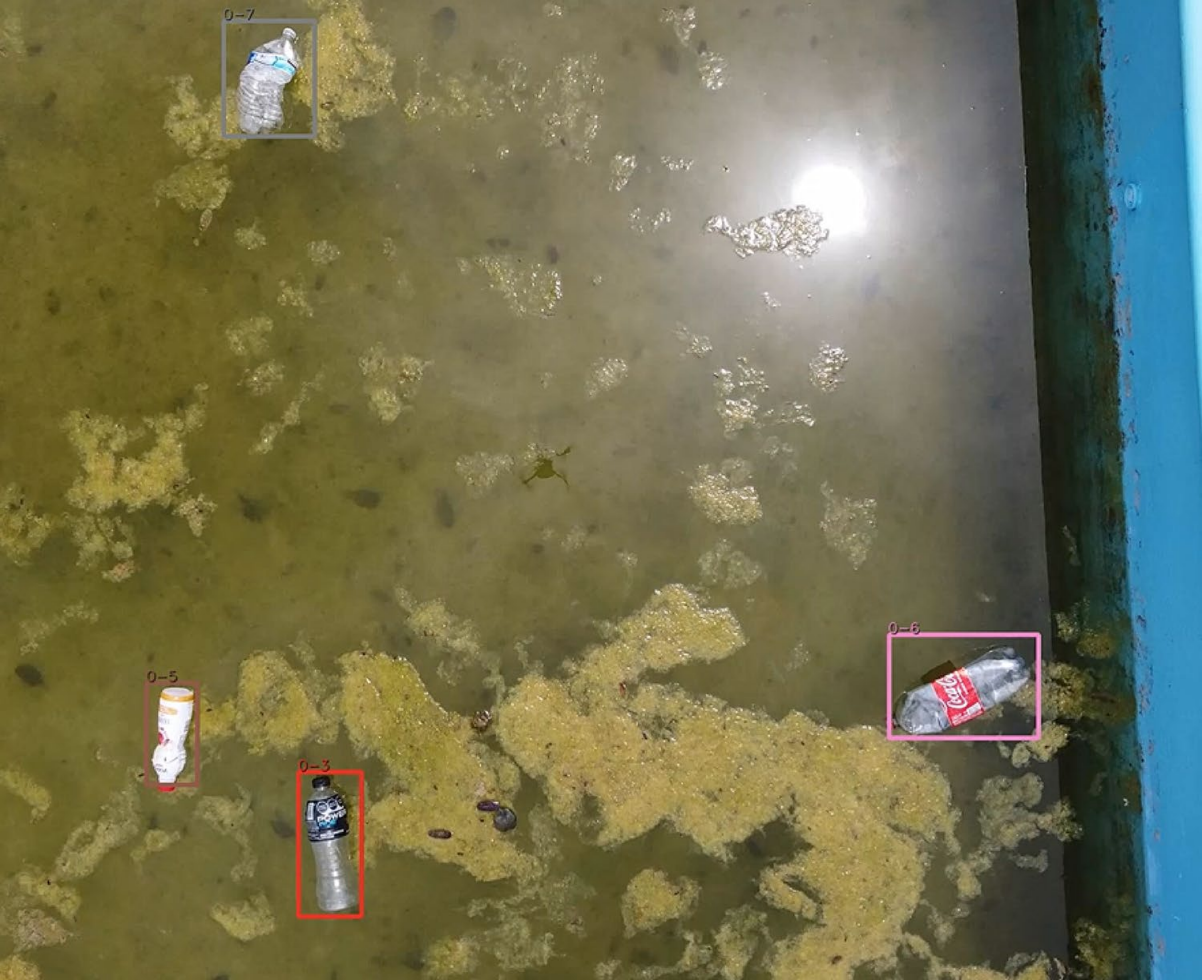
Drone image with detection. Note the large sun reflection during the testing procedures. Sun glare caused detection and tracking issues, resulting in a double count for one bottle.

The last test involved using drone footage tested in a controlled setting in Yucatán, Mexico. Plastic bottles were introduced into a small swimming structure and a drone flying overhead captured continuous video. The model was capable of capturing all of the bottles but struggled due to sun reflections. Of the 6 bottles in the video, the algorithm counted all bottles and double counted one bottle as it passed the sun spot in the water. An image from the test video is shown in Fig. 10. While the recall was 1.0, the precision was limited to 0.88.

The drone footage did not use the object filtering techniques as described in the detection filtering section. This is because the bottle stays in a fixed location in reference to its background.

### Computational Performance

**botell.ai**’s computational performance varies significantly depending on the hardware, with GPU processing being substantially faster than CPU. On a high-performance desktop equipped with an NVIDIA A4000 GPU, the system processes between 30 and 60 frames per second, depending on the video resolution. In contrast, when running on a CPU–specifically a notebook with an Intel i7-1260P (12 cores)–the processing speed drops to approximately 3 frames per second. Although performance can be improved by reducing the input framerate, doing so may compromise accuracy, especially when tracking fast-moving objects.

## Model Use

**botell.ai** is an open source package, written in Python with the Anaconda distribution platform, and is available on GitHub^28^. The model runs in the command line with several options including frame skipping, minimum time an object is on frame, minimum distance traveled, video display for debugging, and detection threshold. After downloading and installing **botell.ai** users can run the following command for help: python bottledetector.py –help.

The application only requires a video path as an input argument and will output a text file with all model inputs as a header and a list of detections, which includes their ID number, confidence, start and end times, and total pixels traveled. A simple simulation would include the following command python bottledetector.py Example.mp4, where Example.mp4 is the name of the sample video.

Each video requires a manual tuning of parameters based on the physical environment (e.g. water velocity, camera distance, and lighting). For long videos, a smaller test segment can be used to determine optimal settings, which can then be applied to the rest of the footage. Additionally, reducing the frame rate and resolution can also help speed up the model.

## Conclusion

**botell.ai** demonstrated excellent performance, accurately detecting, tracking, and counting nearly every bottle during the optimal angle tests (0.95 and 0.98 recall). The sub-optimal angle tests, performed poorly (0.32 and 0.71 recall), and highlighted the importance of video quality (e.g. camera angle, resolution, frame rate, and distance from the object). The drone and side-view tests also showed promising results. There was only one false positive across the six tests due to the filtering algorithm.

Overall, the detection model does not work well on videos with excessive glare, large waves, or distant camera placement. Additional testing was also conducted using TLC2020 timelapse camera. However, the timelapse camera struggled due to low framerate and a lack of continuous tracking over the video.

The **botell.ai** program is a flexible bottle detection model that can be applied across a wide range of environments. Tracking bottles instead of all forms of trash increased the model’s robustness and accuracy, and nearly eliminated false positives. The next steps for this tracking model are to deploy cameras throughout the watershed and track the movement of bottles over storms and extended time periods.

## Data Availability

The datasets supporting the conclusions of this article are available in the CUAHSI’s hydroshare repository, https://www.hydroshare.org/resource/4c6424e5569a4ff89841a7b5b47d31bd/^44^. The software, **botell.ai** v24.10, is publicly available on GitHub and can be accessed with the following link doi: 10.5281/zenodo.13951962^28^. The software is platform independent and is written in Python. It follows the Creative Commons 4.0 Attribution license. The training data, software, and testing videos are all open source and publicly available. The three training datasets are published and accessible from the references^12,14,43^.
